# Effects of Strong Acidic Electrolyzed Water in Wound Healing via Inflammatory and Oxidative Stress Response

**DOI:** 10.1155/2020/2459826

**Published:** 2020-12-12

**Authors:** Ailyn Fadriquela, Ma Easter Joy Sajo, Johny Bajgai, Dong-Heui Kim, Cheol-Su Kim, Soo-Ki Kim, Kyu-Jae Lee

**Affiliations:** ^1^Department of Environmental Medical Biology, Wonju College of Medicine Yonsei University, Wonju, Gangwon 26426, Republic of Korea; ^2^Department of Laboratory Medicine, Wonju College of Medicine Yonsei University, Wonju, Gangwon 26426, Republic of Korea; ^3^Department of Biology, College of Science, University of the Philippines Baguio, 2600, Philippines; ^4^Department of Microbiology, Wonju College of Medicine Yonsei University, Wonju, Gangwon 26426, Republic of Korea; ^5^Institute for Poverty Alleviation and International Development, Yonsei University, Wonju, Gangwon 26493, Republic of Korea

## Abstract

Strong acidic electrolyzed water (StAEW) is known to inactivate microorganisms but is not fully explored in the medical field. This study is aimed at exploring StAEW as a potential wound care agent and its mechanism. StAEW (pH: 2.65, ORP: 1159 mV, ACC: 32.1 ppm) was sprayed three times a day to the cutaneous wounds of hairless mice for seven days. Wound morphological and histological features and immune-redox markers were compared with saline- (Sal-) and alcohol- (Alc-) treated groups. Results showed that the StAEW group showed a significantly higher wound healing percentage than the Sal group on days 2, 4, 5, and 6 and the Alc group on day 4. The StAEW group also showed earlier mediation on proinflammatory cytokines such as tumor necrosis factor-*α*, interleukin- (IL-) 6, IL-1*β*, and keratinocyte chemoattractant. In addition, basic fibroblast growth factor and platelet-derived growth factor were found to be significantly changed in favor of the fibroblast synthesis and angiogenesis. In line, the StAEW group showed a controlled amount of ROS and significantly decreased compared to the Alc group. The StAEW group also favored oxidative stress balance through antioxidant responses. Additionally, matrix metalloproteinases (MMP) 9 and MMP1 were also modulated for keratinocyte and cell migration. Taken together, this study has proven the wound healing effect of StAEW and its earlier mediation through oxidative and inflammatory responses.

## 1. Introduction

Acidic electrolyzed water (AEW) or also known as electrolyzed oxidizing water is produced by a machine through electrolysis of water where a diluted salt solution and water pass through and produces water with oxygen gas, chlorine gas, hypochlorite ion, hypochlorous acid, and hydrochloric acid as components. The general characteristics of acidic water are as follows: low pH (2.2-6.5), high oxidation-reduction potential (ORP) (800-1100 mV), high available chlorine concentration (ACC) (10-60 ppm), and high content of dissolved oxygen [1]. Because of its wide range in pH, researchers in this field came up with the classification of AEW: slightly AEW (SAEW), weak AEW (WAEW), and strong AEW (StAEW). Since the discovery of the production of acidic water, several studies have been conducted over the years that prove its efficacy in killing microbes, fungus, viruses, and inactivating toxins [1]. It has also been studied for its effect on disinfecting food equipment, vegetables, fruits, poultry, and meat; on hand washing; and on hospital bactericidal effect [2–4]. Recent studies in AEW concluded its damage to cell membranes and cell homeostasis of *Pseudomonas fluorescens* biofilms [5] and was also found to enhance the reactive oxygen species (ROS) scavenging capacity of fruits for its enhanced storability [6].

To understand how AEW may potentially facilitate wound healing, it is important to know the wound healing process. Wound healing is a complex, overlapping but systemic mechanism that is strongly controlled for restoring the integrity of the skin. The process consists of four phases namely: haemostasis, inflammation, proliferation, and remodeling phases. Because it is a complex mechanism, several factors are involved such as nutrition, hypoxia, infection, immunosuppression, chronic disease, wound management, age, and genetics [7]. In addition, pH has a great influence in wound healing because of its effect on controlling wound infection, increasing antimicrobial activity, altering protease activity such as matrix metalloproteinases (MMPs) and tissue inhibitor of MMPS (TIMPs), releasing oxygen, reducing toxicity of bacterial end products, and enhancing epithelialization and angiogenesis [8–11]. Thus, there are increasing studies on the use of wound care agents or dressings that control and alter wound pH to accelerate the healing process. The progression of the wound stages depends on the wound type and its pathological condition as well as the type of dressing material. Some of the criteria for dressing selection are its ability to provide protection against bacterial infection, enhance epidermal migration, and promote angiogenesis and connective tissue synthesis, and it must be sterile, nontoxic, and nonallergic [12]. However, the safety of some wound healing agents can still pose a threat due to their possible side effects. StAEW has been reported as generally safe both *in vitro* and *in vivo*. A biocompatibility study of StAEW concluded that acidic water did not show cytotoxicity even in a dose-dependent manner, and a skin irritation test on a human epidermal model reported StAEW as nonirritant [13]. Additionally, another study concluded that electrolyzed waters are generally safe as evidenced by cytotoxicity assay and phospholipid leakage examination [14]. Furthermore, the involvement of the oxidative stress response and immune responses in wound healing has been one of the keys in studying wound care treatment. The good balance of reactive oxygen species (ROS) and antioxidant enzymes also known as redox balance leads to a normal and successful wound healing process [15].

Immunological and oxidative stress regulation has been shown to mediate skin diseases such as atopic dermatitis [16]. Since the development of AEW production, a few studies have been done over the years exploring its function in the medical field. Due to its widely known antibacterial effect, some studies have concluded that it can be effective in wound healing [17, 18]. However, these studies did not fully explore how AEW facilitates cutaneous wound healing. Knowing the different properties and characteristic of this water, and the complexity and factors involved in wound healing, we then hypothesized that StAEW can be an effective wound healing agent, and we tried to find out the mechanism of how StAEW facilitate wound healing by measuring oxidative stress and immune response markers and compared its effect with other potent wound care agents.

## 2. Materials and Methods

### 2.1. Preparation of StAEW, Saline, and Alcohol

StAEW was generated from a water electrolyzing machine (HDR, IONIA Co., Ltd. Bucheon, Korea), wherein tap water and 1% NaCl solution was added to the machine and StAEW was collected in the anode area. Tap water had the properties of pH: 7.62, ORP: 500 mV, and ACC: 0.2 mg/mL, and StAEW had pH: 2.65, ORP: 1159 mV, and ACC: 32.1 mg/mL. Saline (Sal) solution (0.9% NaCl, Life Science Co., Ltd., Dangjin, Korea) and 70% alcohol (Alc) (SK Chemical, Ulsan, Korea) were prepared as control groups.

### 2.2. Animal Groupings

Seven-week-old female hairless mice (18 ± 2 g) were purchased from Orient Bio Inc., Seongnam, South Korea, and were placed in a plastic cage (W 172 mm × D 240 mm × H 129 mm, five mice per cage). First, the mice were acclimatized for one week and kept at room condition of 22°C and 40-60% humidity in 12 : 12 h light and dark. Animals were randomly assigned into four groups with ten mice each: NC: normal control, no wound induction group; and wound-induced groups: Sal: saline-treated groups; Alc: alcohol-treated group; and StAEW: strong acidic electrolyzed water-treated group. Institutional Animal Care and Use Committee (IACUC), Yonsei University Wonju Campus (YWC-170907-1), approved the animal use and protocol done in this study.

### 2.3. Wound Induction and Treatment

To induce the wounds, a total of six wounds were created at the back part of each hairless mouse following protocols [19] by using a 5 mm biopsy punch (Integra-Miltex, PA, USA). Wounds were treated by spraying 2 mL of treatment solutions on the wounded areas, three times a day for seven days.

### 2.4. Wound Area Measurement and Gross Examination

Wound size was measured using a digital caliper vernier scale (Mitutoyo Corp., Japan) to measure the length and width of the two wounds located on the middle part of the mice, and the wound area was computed. Wound healing percentage was measured using the following formula [20]:

(1) $$\begin{array}{c} \text{Wound healing}\%=\left[\left[\text{wound are}{\text{a}}_{\left(\text{day }0\right)}-\text{wound are}{\text{a}}_{\left(\text{day desired}\right)}\right]/\text{ wound are}{\text{a}}_{\left(\text{day }0\right)}\right]\times 100. \end{array}$$

Wounds were observed macroscopically, and a digital photograph was taken every day to check and compare the wound state of each mouse.

### 2.5. Blood Collection and Serum Preparation

Upon sacrifice on the seventh day, blood samples were collected from the retroorbital plexus puncture by using EDTA (Ethylenediamine Tetra-Acetic Acid) and BD Microtainer®. Approximately, blood was collected in each tube per mouse in a microcontainer and used for hematological and biochemical analyzer. Briefly, EDTA microcontainer blood was used for white blood cell (WBC) analysis by mixing thoroughly for 3-5 minutes with an automatic rolling mixer and was counted by hematology analyzer (Hemavet® HV950 FS; Drew Scientific, Erba Diagnostics, Inc., Dallas, Texas, USA). BD Microtainer® blood samples from each mouse were further centrifuged at700 − 1,000 × gfor 10 min at 4°C to get the serum and were kept at -80°C until analysis.

### 2.6. Skin Lysate Preparation

Equal size of skin tissue from the dorsal area was cut and was placed in ice-cold RIPA buffer (Pierce Biotechnology, Inc.) with protease and proteinase inhibitor cocktail (Sigma Chemical Co., St Louis, MO, USA). The crude skin lysate then was homogenized at 25 rpm for 15 min using a Tissue Lyser II (Qiagen, MD, USA). The lysate was then centrifuged for 5 minutes, and the supernatant was collected and transferred to a new epitube and was kept at -80°C until further use. Pierce BCA Assay Kit (Thermo Scientific, Rockford, IL, U.S.A.) was used to check the total protein concentration.

### 2.7. Histological Examination

The representative skin of each group was cut from the wound area and was fixed in 10% buffered formalin (0.1 M phosphate buffer, pH 7.4), dehydrated through a graded ethanol series, cleared by xylene, and embedded in paraffin wax (Polyscience, WA, USA). The paraffin section was cut into 4 *μ*m thick by microtome (Reichert, Inc. New York, USA), stained by hematoxylin and eosin stains, and was observed under the light microscope (BA300, Motic Ltd., Hongkong, China).

### 2.8. WBC and Its Differential Counts Examination

Total WBC, lymphocytes, monocytes, and neutrophil counts were measured by an automatic blood analyzer (HEMAVET HV950 FS, Drew Scientific Inc., Dallas, TX, USA).

### 2.9. Total ROS Detection

Total ROS was measured using DCFH-DA (Abcam, Cambridge, MA, USA) following the manufacturer's manual. In brief, 25 *μ*L of serum and 50 *μ*L of skin lysate were plated into a 96-well plate. Then, 100 *μ*L of 10 *μ*M DCFH-DA was added to the wells, and the plate was incubated in the dark for 30 minutes. Fluorescence reading of the plate at 488 nm excitation/525 nm emission using DTX-880 multimode microplate reader (Beckman Counter Inc., Fullerton, CA, USA) was obtained.

### 2.10. Antioxidant Enzyme Assays

The activity of glutathione peroxidase (GPx) and catalase (CAT) in skin lysate was measured using Biovision kits (Milpitas, CA, USA) following the manufacturer's instructions. In brief, protein concentrations from skin lysate were normalized using Pierce™ BCA Protein Assay Kit by Thermo Scientific (Illinois, USA), the standards of each assay were prepared, the reagents from each assay were mixed in a 96-well microtiter plate, and absorbance was read at 340 nm (GPx) and 510 nm (CAT).

### 2.11. Cytokine and Growth Factor Analysis

Serum cytokines such as interleukin- (IL-) 1*β*, IL-6, tumor necrosis factor (TNF)-*α*, keratinocyte chemoattractant (KC), and growth factors such as platelet-derived growth factor (PDGF) and basic fibroblast growth factor (bFGF) were measured using Bead Array Suspension Multiplex Kit (Bio-Rad, San Diego, CA, USA) according to the manufacturer's instructions. Standard curves for each cytokine were generated using the reference concentrations given in the kit. Standards and samples were read in a multiplex bead suspension array system (Bio-Plex 200, BIO-RAD®, CA, USA).

### 2.12. Western Blot Analysis

The protein expression of MMP1, MMP3, and MMP9 (Abcam, Cambridge, MA, USA) was measured by performing western blotting. The prepared normalized skin lysate was equally loaded and separated by electrophoresis on SDS-polyacrylamide gels. It was then transferred to nitrocellulose membranes which were blocked in 5% skim milk in tris-buffered saline (TBS) containing 0.1% Tween 20 for 1 h at room temperature. After blocking, the membranes were incubated with primary antibodies at 4°C with agitation. It was followed by incubation with horseradish peroxidase-conjugated secondary antibodies for 1 h at room temperature. The blots were developed using the Chemiluminescence Western Blot Detection System (BioSpectrum®600 Imaging System, Upland, CA, USA).

### 2.13. Statistical Analysis

The mean values among the groups were analyzed by the GraphPad Prism version 5.0 software packages (GraphPad, La Jolla, CA, USA) and were compared using one-way analysis of variance (ANOVA) followed by subsequent multiple comparison test (Tukey). A *p* value ≤0.05 was considered statistically significant.

## 3. Results

**Figure 1:**
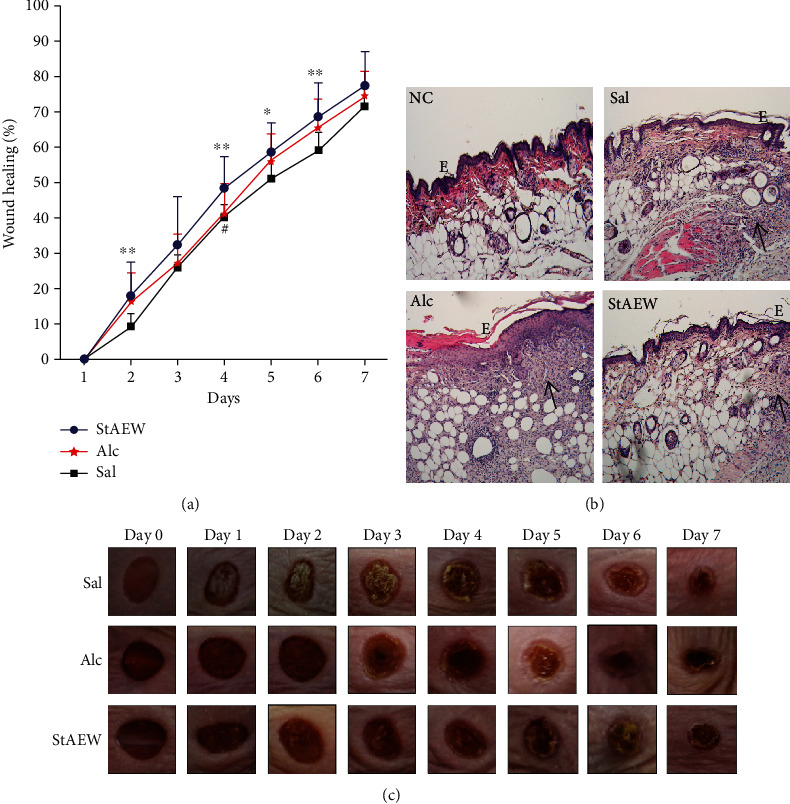
Effects of StAEW on (a) wound healing percentage, (b) wound morphology, and (c) histological appearance from day 1 to day 7 of different treatment groups. Wound size was measured using a digital caliper vernier scale (Mitutoyo Corp., Japan) to measure the length and width of the two wounds located on the middle part of the mice, and the wound area and wound healing percentage were computed. The wounds were observed macroscopically, and a digital photograph was taken every day to check and compare the wound state of each mouse. The histological appearance of cutaneous wounds stained with hematoxylin and eosin. All values are presented as mean ± SD., *n* = 10. ^∗^*p* < 0.05, ^∗∗^*p* < 0.01 vs. Sal group, ^#^*p* < 0.05 vs. StAEW group. StAEW: strong acidic electrolyzed water; Sal: saline; Alc: alcohol.

The effects of StAEW on wound healing percentage and wound morphology and histology wound healing percentage of StAEW were compared with saline (Sal) and alcohol (Alc) treatment on wounded mice. The wound healing percentage of the StAEW group was significantly higher than the Sal group on days 2, 4, 5, and 6 and the Alc group on day 4 (Figure 1(a)). At the end of seven days, the wound healing percentage was highest in the StAEW group as compared to the Sal and Alc groups, which shows that wounds are in 71-77% healed on day 7.  Figure 1(b) showed the morphological change of the representative wounds of mice from each group from day 0 to day 7 of treatment. In the histological analysis, the thickness of the epidermis of the StAEW group was more pronounced similar to the NC group and followed by the Sal group, while the epidermis of the Alc group showed markedly increased proliferation of the epidermis layer (Figure 1(c)). In addition, the presence of immune cells was observed more in the Alc group and lesser in the StAEW group.

### 3.1. Effects of StAEW in Total WBC and Its Differential Counts

We examined the changes in total WBC and its differential counts.  Table 1 shows that compared to the NC group, the Sal (*p* < 0.05) and Alc (*p* < 0.05) groups had reduced total WBC, but there was no statistically significant reduction in the StAEW group. We found that neutrophil counts were similar among all treated groups. However, lymphocyte counts were found to be reduced in the StAEW-treated groups (*p* < 0.05), and the Sal- and Alc-treated groups (*p* < 0.01) as compared to the NC group. Similarly, monocytes were significantly reduced in the StAEW, Sal, and Alc groups (*p* < 0.001) as compared to the NC group.

### 3.2. Effect of StAEW in ROS and Antioxidant Enzymes Generation

**Figure 2:**
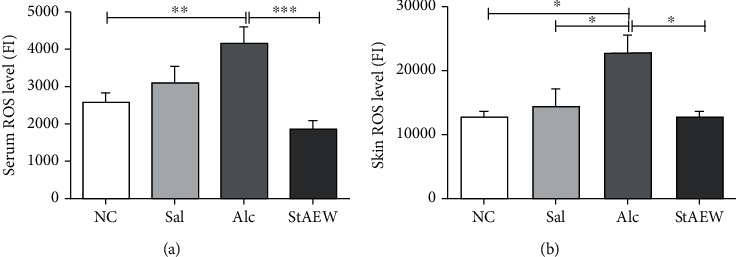
ROS levels in serum (a) and skin lysate (b) among the treatment groups. Total ROS was measured using DCFH-DA. All values are presented as mean ± SD., *n* = 10. ^∗^*p* < 0.05, ^∗∗^*p* < 0.01, and ^∗∗∗^*p* < 0.001. NC: normal control; Sal: saline; Alc: alcohol; StAEW: strong acidic electrolyzed water.

**Figure 3:**
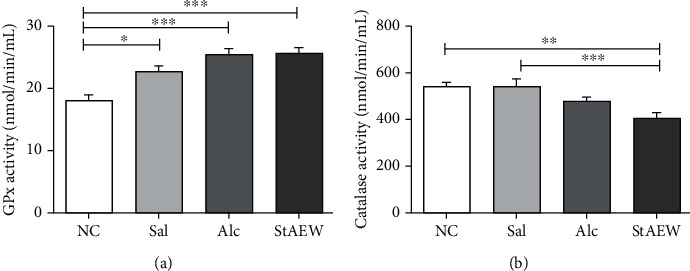
Skin lysate's GPx (a) and catalase (b) levels among the treatment groups. The normalized protein concentration of the skin lysate from each group and standards of each assay were prepared, and reagents were added and were read at 340 nm (GPx) and 510 nm (CAT), respectively. All values are presented as mean ± SD., *n* = 10. ^∗^*p* < 0.05, ^∗∗^*p* < 0.01, and ^∗∗∗^*p* < 0.001 indicate significant differences when tested with ANOVA. Tukey's test was used for post hoc tests. NC: normal control; Sal: saline; Alc: alcohol; StAEW: strong acidic electrolyzed water.

To explore its effect on oxidative stress, the results showed that the ROS level in the serum of the StAEW group had the lowest level of ROS and also showed a significant decrease than the Alc group (*p* < 0.001). In skin lysate, the ROS level showed a similar trend wherein the Alc group consistently have significantly higher ROS level as compared to the NC (*p* < 0.05), Sal (*p* < 0.05), and StAEW groups (*p* < 0.05) (Figure 2). For antioxidant enzyme activities, the GPx level showed a significant increase in the Sal (*p* < 0.05), Alc (*p* < 0.001), and StAEW (*p* < 0.001) groups as compared to the NC group. Catalase activity, on the other hand, was found to be lower in StAEW as compared to the NC (*p* < 0.01) and Sal (*p* < 0.001) groups (Figure 3).

### 3.3. Effects of StAEW in Inflammatory Cytokines

**Figure 4:**
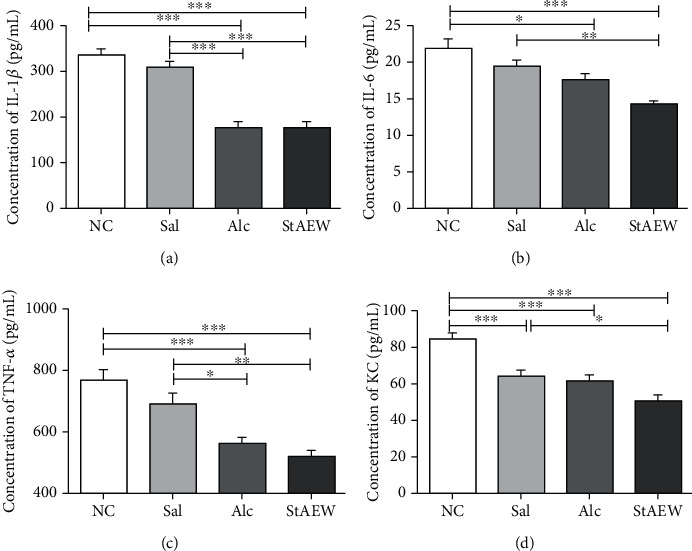
Effect of the treatment groups on IL-1*β* (a), IL-6 (b), TNF-*α* (c), and KC (d). Cytokines were carefully selected and were measured using a multiplex bead suspension array system. All values are presented as mean ± SD., *n* = 10. ^∗^*p* < 0.05, ^∗∗^*p* < 0.01, and ^∗∗∗^*p* < 0.001 indicate significant differences when tested with ANOVA. Tukey's test was used for post hoc tests. NC: normal control; StAEW: strong acidic electrolyzed water.

To explore the mediation of StAEW on various inflammatory mediators such as cytokine production, we measured the concentration of IL-1*β*, IL-6, KC, and TNF-*α*. Our results showed that the IL-1*β*, IL-6, KC, and TNF-*α* concentrations of the StAEW group were significantly decreased as compared to NC (Figure 4). In detail, the IL-1*β* of both the Alc and StAEW-treated groups were significantly reduced as compared to the NC (*p* < 0.001) and Sal groups (*p* < 0.001). On the other hand, only StAEW showed a significant reduction in IL-6 concentration as compared to the NC (*p* < 0.001) and Sal groups (*p* < 0.01). It was also observed that the IL-6 concentration of the Alc group was significantly lower than the NC group (*p* < 0.05). Likewise, TNF-*α* showed a similar trend with IL-1*β* wherein both the Alc and StAEW groups were significantly reduced as compared to the NC group (both *p* < 0.001) and Sal group (*p* < 0.05, *p* < 0.01, respectively). KC concentration showed a similar trend of significant reduction of all treatment groups as compared to NC (*p* < 0.01). A reduction in KC concentration was also observed between the Sal and StAEW groups (*p* < 0.05).

### 3.4. Effects of StAEW in Growth Factors

**Figure 5:**
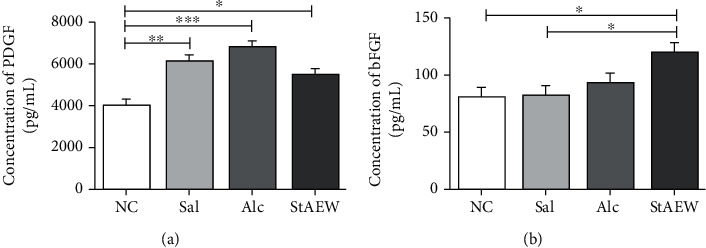
Effect of the treatment groups PDGF (a) and bFGF (b). Growth factors were carefully selected and were measured using a multiplex bead suspension array system. All values are presented as mean ± SD., *n* = 10. ^∗^*p* < 0.05, ^∗∗^*p* < 0.01, and ^∗∗∗^*p* < 0.001 indicate significant differences when tested with ANOVA. Tukey's test was used for post hoc tests. NC: normal control; StAEW: strong acidic electrolyzed water.

We also measured the concentration of PDGF and bFGF and found that they significantly changed as compared to the NC group. As compared to NC, PDGF showed a significantly increased level in the Sal (*p* < 0.01) and Alc (*p* < 0.001) groups but not as much as the increase in the StAEW group (*p* < 0.05). Moreover, a significant increase in the bFGF expression was seen in the StAEW group as compared to the NC and Sal groups (*p* < 0.01) (Figure 5).

### 3.5. Effects of StAEW in MMP Expression

**Figure 6:**
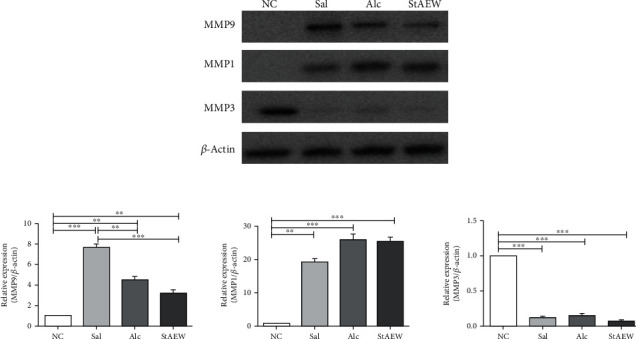
MMP1, MMP3, and MMP9 expressions of the treatment groups. The protein expressions were measured by performing western blotting. Relative intensity was compared to the housekeeping gene, *β*-actin, using ImageJ. All values are presented as mean ± SD., *n* = 10. ^∗∗^*p* < 0.01 and ^∗∗∗^*p* < 0.001 indicate significant differences when tested with ANOVA. Tukey's test was used for post hoc tests. NC: normal control; Sal: saline; Alc: alcohol; StAEW: strong acidic electrolyzed water.

Another important modulator of wound healing is the MMP activity. Our results show that the MMP1 and MMP9 expressions were upregulated in all groups but were not observed in MMP3 as compared to the NC group. The relative expression of MMP9 showed that there was a significant upregulation on the Sal (*p* < 0.001), Alc (*p* < 0.01), and StAEW (*p* < 0.001) groups while the Sal group showed the highest expression and was significantly higher than the Alc (*p* < 0.01) and StAEW (*p* < 0.001) groups. Similarly, the MMP1 expression was also significantly upregulated in the Sal (*p* < 0.01), Alc (*p* < 0.001), and StAEW (*p* < 0.001) groups as compared with the NC group (Figure 6).

## 4. Discussion

Acidic water has been known first to inactivate different microorganisms. We confirmed the efficacy of our experimental water in killing microbes such as *Staphylococcus aureus* and *Pseudomonas aeruginosa* as compared to other kinds of water (Supplementary Figure 1). With this background, this study proved that StAEW could also enhance skin wound healing effects in SKH-1 hairless mice through alleviating inflammatory and oxidative response, thus promoting earlier wound contraction and angiogenesis in wound tissue. This was first supported by the higher wound healing percentage of the StAEW-treated groups after 7 days of treatment especially on the earlier days (days 2 to 6). Our histological results showed that with StAEW treatment, the wounded epidermal area was highly intact than other available conventional wound cleaning agents and showed lesser infiltration of inflammatory cells. Exploring further the possible mechanism of this healing efficacy, it is important to know the involvement of the inflammatory and oxidative response in the wound healing process and its markers.

It is well known that WBCs play an essential role in wound healing and tissue repairing process especially in the earlier stage of wound healing. Upon injury, inflammatory cells migrate into the injury area and promote the inflammatory phase, which is further exemplified by the sequential but overlapping infiltration of neutrophils, macrophages, and lymphocytes [21, 22]. In our study, the reduction of the WBC counts especially the lymphocytes and monocytes indicates the mediation of the treatment groups in wound healing. This mediation of StAEW was also supported histologically by the presence of lesser inflammatory cells at this wound stage compared to other groups.

To continue exploring the immune and oxidative stress response in wound healing, we also explored the oxidative stress response mediated by StAEW. A controlled amount of ROS is well-studied to play an important role in a normal wound-healing response. Some of their roles include actions as the host's defense through phagocytosis, acting as second messengers to immune cells and also regulating angiogenesis [23]. This controlled amount of ROS is attained by the antioxidant's role in scavenging ROS, in a process called redox homeostasis [15]. Consistently, the controlled amount of ROS upon the StAEW treatment in both serum and wound area may suggest its beneficial mediation on the wound healing process, while the significant increase of ROS in both serum and skin lysate in Alc-treated group may suggest its oxidative stress damage upon its treatment. Overproduction of ROS causes oxidative stress thereby causing cytotoxicity and delayed wound healing process, and elimination of these contributing ROS could be an important strategy in wound healing. To support this, the production of antioxidant enzymes seems to be mediated earlier by StAEW. It is essential to note that the functions of antioxidant enzymes in wound healing are in a temporal and spatial expression pattern [24]. Hence, the estimation of antioxidant enzymes like GPx and CAT in granulation tissues is relevant because the antioxidant activities have been reported to accelerate wound healing effect through decreasing the free radicals [25–27]. Therefore, the antioxidant levels obtained after StAEW treatment, along with reduced total ROS, may seem to prevent oxidative damage and promote the healing process.

To further confirm the mediation of StAEW in immune response, cytokines and growth factors involved in the healing process were screened and assayed. Upon careful screening, cytokines, IL-1*β*, IL-6, TNF-*α*, and KC were investigated for the mediation of the treatment groups in response to wound healing. Proinflammatory cytokines IL-1*β*, IL-6, and TNF-*α* are released by neutrophils, macrophages, and keratinocytes and trigger inflammatory cell proliferation and are also involved in promoting angiogenesis [28]. Moreover, the significant change of a chemokine, KC, may attribute to its function on the epithelialisation, angiogenesis, and tissue remodelling [29]. The significant reduction of these cytokines in the StAEW-treated groups shows the potential earlier mediation of StAEW in these cytokines to facilitate the healing process. Our results showed that the Alc-treated groups have a similar effect with StAEW on these cytokines; however, a more decreasing trend was observed in the StAEW groups. In addition to cytokines, growth factors also play essential roles in accelerating the healing process. At this wound stage, mediation of StAEW in PDGF, which is important in the earlier stage of wound healing and functions in inflammation, granulation tissue formation, reepithelialisation, matrix formation, and remodelling [30], was less as compared to the Sal and Alc groups. On the other hand, bFGF, which plays an important role in the late remodelling stage and functions in angiogenesis and granulation tissue formation [31], showed to be significantly mediated by StAEW. Our results revealed that at this wound stage, StAEW seems to mediate the decreased production of PDGF and increased production of bFGF faster than the other conventional wound healing agents.

During the late proliferation and remodelling stages of wound healing, MMPs and their inhibitors play a critical role in irreversible remodelling wherein MMPs mainly degrade substances in the extracellular matrix. Recently, MMPs are known to be also responsible in inflammation, epithelial repair, and resolution. It is also important to note that there is a specific location and wound stages that MMP actions are upregulated and downregulated [32]. Our results showing the upregulation of MMP1 and MMP9 protein expression in wounded mice groups and the downregulation of MMP3 expression may imply the wound stage upon mice sacrifice. It is known that MMP9 produced by keratinocytes at the wound edge facilitates cell migration and reepithelialisation and is also involved in angiogenesis through the activation of TNF-*α* and VEGF [32] while MMP1 facilitates keratinocyte migration [33]. Moreover, MMP3 is known to be not necessary for keratinocyte proliferation or migration, collagen synthesis, and remodelling of the extracellular matrix but has a significant effect on wound contraction [34]. Our results showing the upregulation of MMP9 and MMP3 proves the mediation of StAEW on these proteins, and its decreasing level may suggest its faster mediation on these MMPs than in the Sal and Alc groups.

Taken together, it is certain that along with the known and potent antiseptic agents, such as Sal and Alc, StAEW is not only shown to be effective but also shows its earlier mediation in the wound healing process. This is done through its mediation on the immune response, as evidenced by the lesser inflammatory cells, the reduction of inflammatory cytokine and wound-stage-dependent growth factor release, and oxidative response, through providing a redox environment essential to the wound healing process, shown by the controlled amount of ROS and the significant difference of antioxidant enzymes and also through the crosslink of wound-stage-dependent MMP expressions. As more evidences and studies prove the efficacy of StAEW not only in killing microorganisms but also in the wound healing process, it is important to make sure that StAEW is retained in the wound site. One way is by freshly preparing StAEW and the repeated applications on the wound site. Our study showed that application three times a day was found effective. However, further studies might be necessary to check on the frequency of application as well as the possible use of gauzes or the development of hydrogels to retain the StAEW. However, as a recent report showed that physical properties such as pH, ORP, and available chlorine concentration of electrolyzed waters might be affected by storage, temperature, and light exposure [35], it is important to make sure that the StAEW is prepared and stored well to maximize its full healing efficacy. In conclusion, StAEW can be used as an antiseptic agent in wounds and as a wound dressing. This result is consistent with the previous study that our lab reported on the immune-redox modulation of slightly acidic water (pH: 5–6.50, ORP: 800 mV, ACC:25 ppm) on cutaneous wounds [36]. Over the years, several disinfectants such as Alc and Sal have been used in wound care agents. However, due to its compound chemical properties, it may be possible that these agents can have side effects. The advantages of using StAEW as wound dressing include its faster healing efficacy than Alc and Sal. Moreover, because acidic water has been generally recognized as a safe compound as shown in the biocompatibility studies [13, 14], including toxicity and mutagenicty study in wound healing [35] and even on the application on the skin [37], the use of this agent in wound care can be more advantageous and better alternative to harsh chemical disinfectants. In addition, StAEW is also well-known for its cost-effectiveness due to its economical set-up generation using salt and tap water.

More studies are needed to explore the crosslink of immune response and inflammation factors and other potential parameters such as gender and use of other mice strains, which may affect the earlier mediation of StAEW in the healing process. An in-depth study of checking the effect of StAEW on each wound stage would be a great future study to fully understand its effect in wound healing responses and to take full advantage of the application of StAEW.

## Figures and Tables

**Table 1:** Total white blood cells (WBC) and its differential counts.

WBC count (K/*μ*L)	NC	Sal	Alc	StAEW
Total WBC	5.14 ± 0.95	4.04 ± 0.40^∗^	4.02 ± 0.63^∗^	4.54 ± 0.55
Neutrophils	1.50 ± 0.28	1.66 ± 0.13	1.77 ± 0.39	1.80 ± 0.28
Lymphocytes	3.24 ± 0.80	2.12 ± 0.34^∗∗^	2.04 ± 0.39^∗∗^	2.47 ± 0.48^∗^
Monocytes	0.41 ± 0.09	0.26 ± 0.07^∗∗∗^	0.19 ± 0.05^∗∗∗^	0.22 ± 0.05^∗∗∗^

Data were expressed as mean ± SD, *n* = 10. NC: normal control; StAEW: strong acidic electrolyzed water. ^∗^*p* < 0.05, ^∗∗^*p* < 0.01, and ^∗∗∗^*p* < 0.001 compared with the NC group indicate significant differences with ANOVA. Tukey's test was used for post hoc tests.

## Data Availability

Data will be provided upon request to the corresponding author.

## Supplementary Materials

Supplementary Materials Supplementary Figure 1. Staphylococcus aureus (A) and Pseudomonas aeruginosa (B)-culture treated with different kinds of water. PW: purified water; TW: tap water; DW: distilled water; StAEW: strong acidic electroyzed water.
