# Supplementary material for: Effects of Strong Acidic Electrolyzed Water in Wound Healing via Inflammatory and Oxidative Stress Response
Source: Oxid Med Cell Longev. 2020 Dec 12;2020:2459826. doi: 10.1155/2020/2459826 (PMC7752269; doi:10.1155/2020/2459826)
Supplement: Supplementary Materials — Supplementary Figure 1. Staphylococcus aureus (A) and Pseudomonas aeruginosa (B)-culture treated with different kinds of water. PW: purified water; TW: tap water; DW: distilled water; StAEW: strong acidic electroyzed water. [file 2459826.f1.pdf]

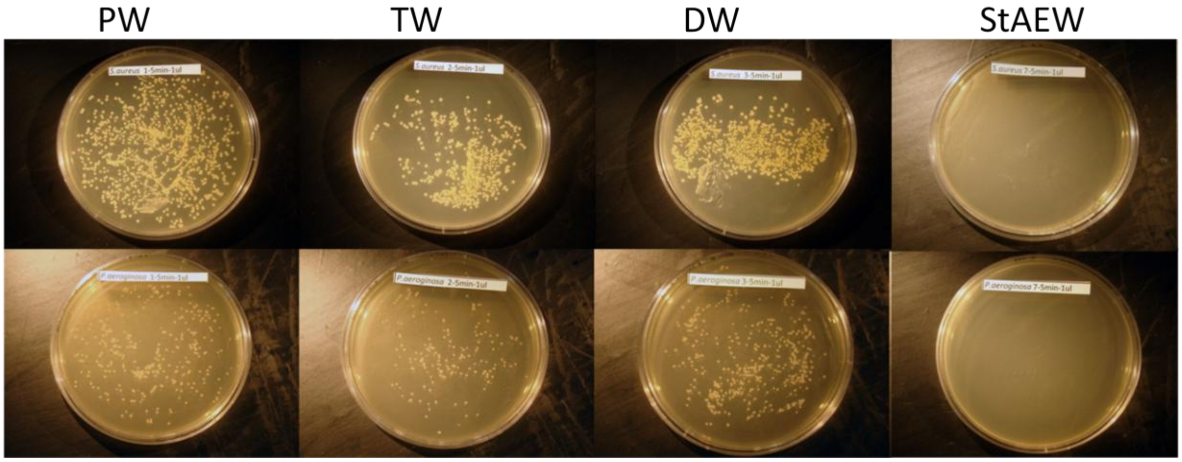

Supplementary Figure 1. *Staphylococcus aureus* (A) and *Pseudomonas aeroginosa* (B)-culture treated with different kinds of water. PW: purified water; TW: tap water; DW: distilled water; StAEW: strong acidic electroyzed water
